# Doctors taking bribes from pharmaceutical companies is common and not substantially reduced by an educational intervention: a pragmatic randomised controlled trial in Pakistan

**DOI:** 10.1136/bmjgh-2024-016055

**Published:** 2025-01-15

**Authors:** Mishal Khan, Muhammad Naveed Noor, Afifah Rahman-Shepherd, Amna Rehana Siddiqui, Sabeen Sharif Khan, Nina van der Mark, Afshan Khurshid Isani, Ahson Q Siddiqi, Charles Opondo, Faisal Ziauddin, Faiza Bhutto, Iqbal Azam, Johanna Hanefeld, Natasha Ali, Robyna Irshad Khan, Syed Ahmed Raza Kazmi, Virginia Wiseman, Wafa Aftab, Zafar Mirza, Zainab Hasan, Sameen Siddiqi, Rumina Hasan, Sadia Shakoor

**Affiliations:** 1 Faculty of Public Health and Policy, London School of Hygiene and Tropical Medicine, London, UK; 2 Department of Pathology and Laboratory Medicine, Aga Khan University, Karachi, Pakistan; 3 Department of Community Health Sciences, Aga Khan University, Karachi, Pakistan; 4 Department of Community Health Sciences, University of Manitoba, Winnipeg, Manitoba, Canada; 5 Jinnah Sindh Medical University, APPNA Institute of Public Health, Karachi, Pakistan; 6 Health Department, Government of Sindh, Karachi, Pakistan; 7 Sindh Healthcare Commission, Karachi, Pakistan; 8 Department of Medical Statistics, London School of Hygiene and Tropical Medicine Faculty of Epidemiology and Population Health, London, UK; 9 Nuffield Department of Population Health, University of Oxford, Oxford, UK; 10 Department of Medicine, Ziauddin University, Karachi, Pakistan; 11 Department of Anaesthesiology, Aga Khan University, Karachi, Pakistan; 12 The Kirby Institute, University of New South Wales, Sydney, New South Wales, Australia; 13 Department of Global Public Health and Primary Care, University of Bergen, Bergen, Norway; 14 School of Universal Health Coverage, Professor of Health Systems at Shifa Tameer-i-Millat University, Islamabad, Pakistan; 15 Faculty of Infectious and Tropical Diseases, London School of Hygiene and Tropical Medicine, London, UK

**Keywords:** Public Health, Health systems

## Abstract

**Introduction:**

Incentive-linked prescribing, which is when healthcare providers accept incentives from pharmaceutical companies for prescribing promoted medicines, is a form of bribery that harms patients and health systems globally. We developed a novel method using data collectors posing as pharmaceutical company sales representatives to evaluate private doctors’ engagement in incentive-linked prescribing and the impact of a multifaceted educational intervention on reducing this practice in Karachi, Pakistan.

**Methods:**

We made a sampling frame of all doctors running for-profit, primary-care clinics and randomly allocated participants to control and intervention groups (1:1). The intervention group received a multifaceted seminar on ethical prescribing and reinforcement messages over 6 weeks. The control group attended a seminar without mention of ethical prescribing. The primary outcome was the proportion of participants agreeing to accept incentives in exchange for prescribing promoted medicines from data collectors posing as pharmaceutical company representatives, 3 months after the seminars.

**Results:**

We enrolled 419 of 440 eligible participants. Of 210 participants randomly allocated to the intervention group, 135 (64%) attended the intervention seminar and of 209 participants allocated to the control group, 132 (63%) attended the placebo seminar. The primary outcome was assessed in 130 (96%) and 124 (94%) of intervention and control participants, respectively. No participants detected the covert data collectors. 52 control group doctors (41.9%) agreed to accept incentives as compared with 42 intervention group doctors (32.3%). After adjusting for doctors’ age, sex and clinic district, there was no evidence of the intervention’s impact on the primary outcome (OR 0.70 [95% CI 0.40 to 1.20], p=0.192).

**Conclusions:**

This first study to covertly assess deal-making between doctors and pharmaceutical company representatives demonstrated that the practice is strikingly widespread in the study setting and suggested that substantial reductions are unlikely to be achieved by educational interventions alone. Our novel method provides an opportunity to generate evidence on deal-making between doctors and pharmaceutical companies elsewhere.

**WHAT IS ALREADY KNOWN ON THIS TOPIC**

- Healthcare providers making hidden deals to take incentives from pharmaceutical companies for prescribing promoted medicines is common in a range of countries, including Pakistan, and is detrimental to patients and health systems.

**WHAT THIS STUDY ADDS**

- This is the first study to evaluate private doctors’ hidden engagement in incentive-linked prescribing deals using standardised sales representatives (data collectors posing as sales representatives of a fictitious pharmaceutical company).
- We found that willingness to accept incentives to prescribe is strikingly common and that a multifaceted educational intervention did not significantly reduce doctors’ deal-making.
- Our study also revealed that doctors played an active role in negotiating a range of incentives. Those who declined to make deals with our covert data collector typically did so for reasons other than an ethical objection to incentive-linked prescribing, such as already having too many deals with other companies.

**HOW THIS STUDY MIGHT AFFECT RESEARCH, PRACTICE OR POLICY**

- Our findings suggest that deal-making between doctors and the pharmaceutical industry requires greater attention as an impediment to quality healthcare, and calls into question the impact of educational-based approaches alone in shifting doctors towards more ethical prescribing practices.
- Our novel covert method was effective at assessing interactions between doctors and pharmaceutical sales representatives and may be a useful tool to provide empirical evidence on incentive-linked prescribing for monitoring and regulation.

## Introduction

 The pharmaceutical industry’s influence on prescribing of medicines is unequivocal. It is well documented that pharmaceutical companies use a range of approaches, including bribery, to incentivise prescribing of promoted medicines in numerous countries.^1^,^4^ Deals between healthcare providers and pharmaceutical companies create a conflict of interest because the providers’ professional judgement concerning a primary interest (the patient’s welfare) is at risk of being unduly influenced by a secondary interest (financial gain).^5^ We use the term incentive-linked prescribing to refer to situations in which healthcare providers accept incentives from pharmaceutical companies in return for prescribing medicines specified by the company. Incentive-linked prescribing is known to drive the prescription of unnecessary or overly expensive medicines; this results in higher costs for patients, with a differential impact on the poorest, increases patients’ risks from adverse effects, and undermines trust in the healthcare system.^16^,^8^ Qualitative data from a diverse range of countries, including Pakistan, Lebanon, China and America indicate that deal-making between doctors and pharmaceutical company representatives for prescribing medications has become normalised practice.^9^,^12^ However, because interactions in which incentives are offered to healthcare professionals are typically hidden, there is a dearth of robust evidence quantifying how common this practice is and how to reduce it.^1 3^

Investments in educational approaches that enhance doctors’ awareness of appropriate interactions with the pharmaceutical industry are widely advocated^8^. Educational approaches may also help garner doctors' support for measures aimed at curtailing practices from which they benefit financially, including implemention of stronger regulatory measures that limit incentive-linked prescribing. Longstanding deficiencies in compulsory medical education and guidelines pertaining to ethical prescribing and management of conflicts of interest that arise from healthcare providers’ interactions with the pharmaceutical industry are problematic, particularly in low and middle-income countries with healthcare sectors dominated by insufficiently regulated for-profit entities.^13 14^ A notable illustration is the static nature of the WHO guidelines on ethical criteria for drug promotion, which have not been updated since they were introduced in 1988.^15^

However, while there is evidence to suggest that careful framing of messages about incentive-linked prescribing may influence doctors’ attitudes and practices,^16^,^18^ studies from around the world illuminate numerous deep-rooted drivers that make it difficult to reduce deal-making between doctors and the pharmaceutical industry.^28 19^,^23^ These drivers include increasing commercialisation of medical practice, normalisation of corruption in society, and patient demand for multiple medications for (perceived) faster recovery.^24^ The impact of education-based approaches on substantially reducing doctors’ deal-making with pharmaceutical companies is therefore uncertain and warrants investigation.

In this study, we implement a novel method to covertly evaluate how common it is for private doctors to engage in incentive-linked prescribing deals and evaluate the impact of a multifaceted education-based intervention on incentive-linked prescribing practices, knowledge and attitudes.

## Methods

### Study design

We conducted a parallel single-blinded placebo-controlled randomised trial in Karachi, Pakistan. Karachi has over 20 million residents^25^ and extremely limited public primary healthcare services.^26^ Documentation of the sprawling private healthcare sector is incomplete; approximately 2000 allopathic primary care clinics and several thousand untrained healthcare providers operate in the city.^27^ Further details on the study setting are in box 1. The study protocol is available online.^28^

Box 1 **Study setting**

Pakistan is the fifth most populous country in the world. Over 70% of healthcare utilisation happens in the private sector, and funding for public services has been declining since the 1990s.^27^ Karachi is Pakistan’s largest city, and this is reflected in the size of the healthcare market.^40^ Small-scale for-profit clinics, such as the ones we include in this study, constitute a major part of the healthcare sector in urban areas of Pakistan.

Pakistan’s health system is characterised by a rapidly growing pharmaceutical industry, and it faces challenges with both oversupply and inadequate access to medicines.^41^ Approximately 700 pharmaceutical companies operate in Pakistan, of which less than 30 are multinational companies.^42^ International industry guidelines by the WHO and the International Federation of Pharmaceutical Manufacturers and Associations are not widely followed by companies in Pakistan.^42^ At a national level, the Drug Regulatory Authority published rules for ethical marketing in the health sector in 2021,^30^ and the Pakistan Medical and Dental Council published a code of ethics for doctors in 2002.^43^ Under these policies, healthcare professionals may not accept, and pharmaceutical companies cannot offer, any cash, food, gift baskets, flowers or other branded promotional goods to influence professionals in the medical, dental, pharmacy or nursing professions or to anyone involved in recommending, prescribing, buying, supplying, dispensing or administrating pharmaceutical products. Despite these policies, there is widespread evidence that incentive-linked prescribing is a ubiquitous and normalised practice in Pakistan.^21 41 42 44 45^ There are no accredited certification courses, awareness sessions or formal pledges required of doctors to comply with the Pakistan Medical and Dental Council code of ethics. Qualitative analyses indicate that both multinational pharmaceutical companies and local manufacturing companies may engage in drug promotion activities that involve incentivisation of doctors to influence prescribing practices.^19^

### Participants

To identify eligible participants, we produced a verified sampling frame of all private doctors working in for-profit, primary healthcare clinics in any of Karachi’s six districts, using the provincial regulator (Sindh Healthcare Commission) database. The database contained 1031 potentially eligible participants after excluding duplicate and erroneous entries, and we checked all of these for eligibility. We excluded clinics run by providers who did not have a medical license, who worked in non-profit clinics or who offered specialist rather than primary care. We first applied our inclusion and exclusion criteria to the information documented in the Sindh Healthcare Commission database and then called and visited potentially eligible providers to validate their information against our eligibility criteria. When clinic numbers listed in the regulator’s database were not answered after three calls over three different days and times, we classified them as unreachable. Eligible private doctors were invited to participate in their clinics. Written consent to participate in the study, including the ‘unannounced assessment’ of their practice, was taken by a research team member.

### Randomisation and masking

The trial statistician (IA) worked independently to randomly assign all enrolled private doctors, stratified by district, to either the intervention or control group (1:1) using an allocation sequence generated in Microsoft Excel. Complete masking of participants was not possible owing to the nature of the intervention. However, all participants received the same hard copy invitation to the seminar, which was titled ‘Professional Development Seminar on Overcoming Challenges in the Delivery of Primary Care’, and information provided during enrolment did not mention ethical practice or conflict of interest. Steps were also taken to ensure appropriate masking of the research team. Participants were assigned unique identification numbers which did not include any information about their allocation to the intervention or control group, and members of the study team involved in documenting and assessing outcomes of the intervention were masked to group allocations until outcomes were determined.

### Intervention and procedures

**Figure 1:**
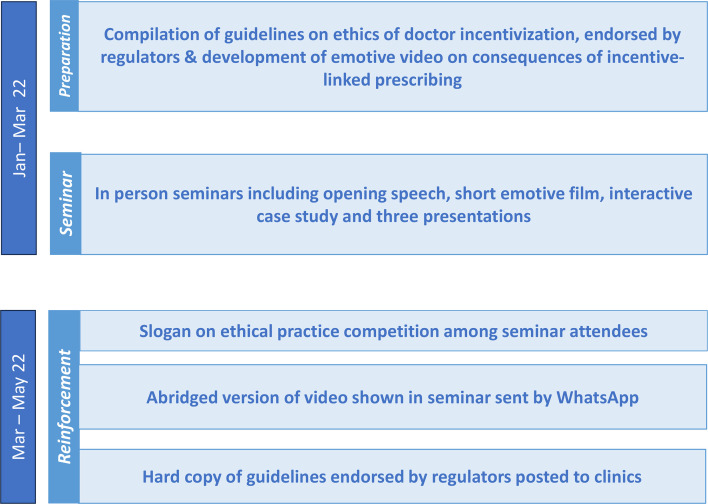
Visual summary of intervention procedures.

Participants in the intervention and control groups attended a single in-person seminar in March 2022. To maximise attendance at the seminars, there were two seminar dates to choose from, all consenting private doctors were sent hard-copy invitations (January 2022), followed by reminders via WhatsApp (February 2022) and we made reminder telephone calls in the week leading up to the seminars. A short visual summary of the procedures is shown in figure 1.

The intervention group received a seminar and three reinforcement messages over the 6 weeks that followed the seminars (see online supplemental materials). Intervention seminars, informed by 12 months of formative research,^2 10^ included:

1. An opening speech about the need for the medical community to live up to the trust placed in it by society, delivered by a clinician experienced in teaching professional medical ethics.
2. A short film developed by the study team in collaboration with a local media company, illustrating how incentive-linked prescribing can adversely impact patient and physician well-being.
3. A case study in which participants discussed a range of consequences related to incentive-linked prescribing.
4. Three short presentations delivered by: an ethics expert who summarised key policies and guidelines on ethical prescribing practice; a former Minister of Health and well-known columnist who warned about the risks of incentive-linked prescribing to the medical community; and a representative from the provincial regulator who emphasised the seriousness with which the provincial regulator is addressing doctors’ violation of ethical prescribing guidelines.

Reinforcement messages were sent to intervention seminar participants with the aim of continuing their engagement with content conveyed during their seminar. The first involved a competition to design a slogan that would motivate doctors towards more ethical prescribing practices. Participants submitted their slogans to the research team and a panel selected the best ones to print on a certificate of attendance distributed as part of the third message. The second was an abridged version of the film shown in the seminar, highlighting the perils of incentive-linked prescribing. Both of these were shared through WhatsApp. The third was a hard-copy document sent to the participants’ clinic, which included an official letter from the provincial regulator motivating doctors towards more ethical prescribing practices, a 12-point summary of existing guidelines on conflicts of interest (see online supplemental materials), a copy of the latest notification from the Drug Regulatory Authority of Pakistan on ethical marketing to healthcare professionals and a certificate of attendance to the seminar.

Participants in the control group attended a ‘placebo’ professional development seminar of the same length and format, and which focused on sharing guidelines and the best practices on managing blood disorders in primary care, without any mention of ethical prescribing.

### Data collection and outcomes

Demographic information, including sex (female/male/prefer not to answer/prefer to self-describe), was collected in-person using an electronic questionnaire, immediately following consent to participate.

Primary outcome data were collected approximately 3 months after the seminars, between May and mid-June 2022, using a novel approach developed for this study. This approach is detailed in an earlier paper^28^ and in box 2. Briefly, we trained data collectors to present to participants as representatives of a fictitious new pharmaceutical franchise company and to provide information about incentives being offered to doctors who agreed to prescribe their promoted medicines (which included vitamins, antibiotics, a proton-pump inhibitor and a cough syrup). These data collectors posing as pharmaceutical company sales representatives used a standardised script and had a maximum of two meetings with each participant to receive a response. We designed a questionnaire in Urdu for standardised sales representatives to document participant responses during each interaction. This was completed immediately after leaving the participant’s clinic. They additionally summarised each interaction in a voice recording.

Box 2 **Summary of the standardised sales representatives training and methodology**

The 15-data collectors who were recruited by the study team received extensive (2 weeks) training by members of the research team who developed the standardised sales representatives methodology.^28^

The standardised script used by data collectors posing as sales representatives to interact with private doctors, which they memorised and were tested on, had three parts: introduction about their fictitious franchise-model pharmaceutical company that has recently started operations in Karachi; information about the pharmaceutical products they are asking doctor to prescribe; and different types of incentives (clinic equipment, a leisure trip for them and their family; cash or cheque payment) they are able to offer the doctor in exchange for prescribing their promoted medicines.^31^ Each interaction was executed by two data collectors; one acted as a sales representative while the other acted as a sales manager. Doctors were free to select from the incentives mentioned by the standardised sales representative or request a different incentive (such as a meal out with their family paid for by the pharmaceutical company).

Immediately following the interaction, standardised sales representatives completed a prepiloted electronic questionnaire and voice recording. The questionnaire was designed for standardised sales representatives to document participants’ responses based on the standardised script, and the purpose of the voice recording was for standardised sales representatives to verbally describe the outcome of their interaction to ensure consistency with the questionnaire data. All data collected by the standardised sales representatives were cross-checked by MNN and two research supervisors who met with the standardised sales representatives after each interaction to discuss any issues.

The primary outcome was the proportion of participants who agreed to accept one or more incentives in exchange for prescribing medicines promoted by the standardised sales representative. Based on data from the standardised sales representatives’ completed questionnaires and voice recordings, masked members of the study team classified participants’ responses into one of two categories: (1) agreed to accept one or more incentives from the standardised sales representative after two interactions, or (2) did not agree to an incentive from the standardised sales representative after two interactions. The second category included participants who directly declined the standardised sales representative’s offerings, instructed their clinic receptionist to turn away all pharmaceutical company representatives, and did not engage in discussions with the standardised sales representative during the two meetings. The questionnaire completed by standardised sales representatives also captured the type of incentives participants agreed to accept, and reasons why a participant did not agree to an incentive-linked prescribing deal.

Secondary outcomes were assessed by using true or false statements on: knowledge of conflict of interest issues related to pharmaceutical incentivisation (three questions), knowledge of national guidelines on ethical prescribing (four questions) and attitudes towards accepting different types of incentives linked to prescribing targets (seven questions). Secondary outcome data were collected using an electronic questionnaire administered by data collectors in Urdu at the participant’s clinic. Questions were formulated based on a review of existing national guidelines on ethical prescribing and findings from our formative research and were finalised in collaboration with a Pakistani medical ethics expert (RIK).^10 15 29 30^ Further detail on the questions used can be found in the online supplemental materials. Secondary outcome data were collected in June 2022, approximately 2 weeks after primary data collection had been completed.

For primary and secondary outcome data collection, we allowed a maximum of three unsuccessful visits at clinics (defined as visits in which no contact could be made to collect data) before participants were considered loss to follow-up.

We also conducted in-person semistructured interviews with 28 intervention group participants between August and September 2022. Interviewees were purposively selected to represent doctors who did and did not agree to deals with the standardised sales representatives. The interviews explored which content from our intervention was remembered and why it might have or have not impacted practices. Responses were analysed using deductive coding. All data collected in Urdu were translated into English prior to analysis and independently verified for translation accuracy.

### Statistical analysis

We planned to invite all eligible private doctors in the study area to participate in the trial and estimated that two-thirds would attend the seminar at a convenient location. A sample size of 130 participants in each arm would give us 80% power at the 5% level of significance to detect a 15% absolute reduction in the proportion of participants in the intervention arm agreeing to accept one or more incentives. This assumes 30% of participants in the control arm agree to accept one or more incentive and allowing for 5% loss-to-follow-up between seminar attendance and standardised sales representative assessment.

We estimated crude ORs for the primary outcome with 95% CIs using ordinary logistic regression. We also estimated adjusted ORs controlling for participants’ age, sex and district where their clinic is located. For secondary outcomes, we estimated the absolute mean difference in scores between the intervention and control groups using linear regression. We further summarised the types of incentives that private doctors agreed to accept, and the reasons participants did not accept an incentive using frequency counts.

The outcomes analysis includes all private doctors who attended one of the seminars offered. We used STATA V.17.0 for all statistical analyses. This study is registered with the ISRCTN registry (ISRCTN12294839).

### Role of the funding source, patients and the public

This study was funded by a Health Systems Research Initiative grant (MR/T02349X/1). The funder had no role in the design, data collection, analysis and interpretation of the study data nor the report writing or submission process. It was not appropriate or possible to involve patients or the public in the design, or conduct, or reporting, or dissemination plans of our research.

## Results

**Figure 2:**
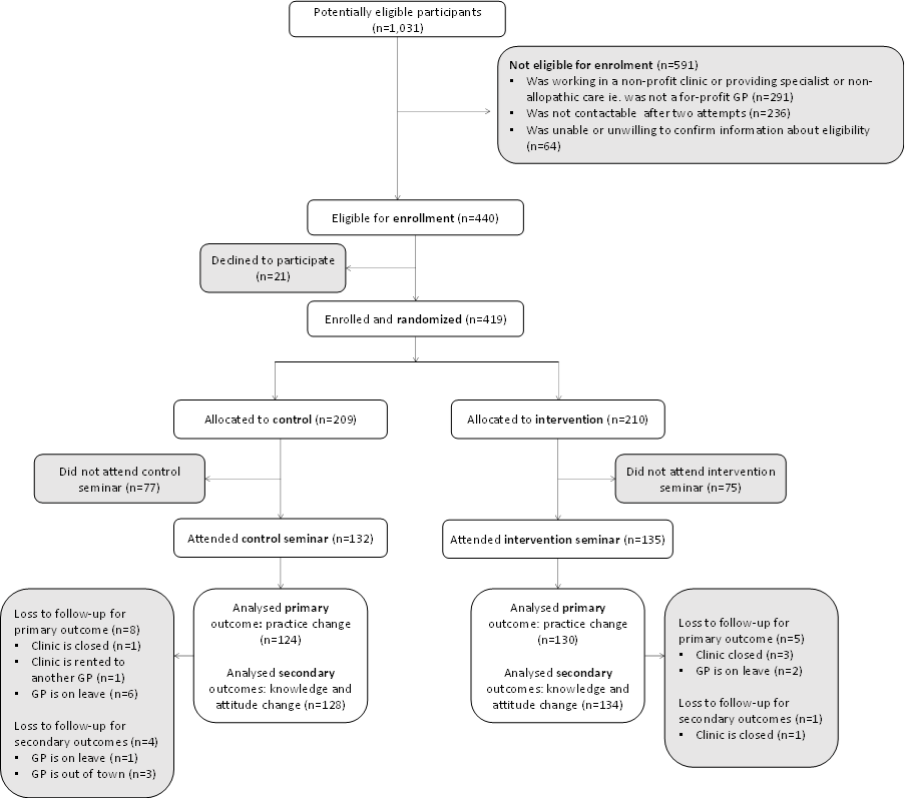
Trial profile. GP, general practitioner.

Of 1031 potentially eligible participants, 291 (28%) were ineligible because they provided specialist or non-allopathic care or worked in non-profit facilities. A further 300 were ineligible owing to a range of reasons detailed in the trial profile (figure 2). Of the 440 eligible private doctors we approached, 21 (5%) refused to participate, resulting in 419 participants being enrolled between 14 October and 1 December 2021. We randomised 210 to the intervention group and 209 to the control group. Of those allocated to the intervention group, 135 (64%) attended the intervention seminar and of those allocated to the control group, 132 (63%) attended the placebo seminar. Of the 135 intervention group doctors invited to participate in the slogan competition, 30 submitted slogans they had designed. We were able to access 96% (n=130) and 94% (n=124) of the intervention and control participants, respectively, for the assessment of the primary outcome. Characteristics of participants in the two groups were similar (table 1).

**Table 1:** Demographics and characteristics of the study sample

	Intervention group (n=135)	Control group (n=132)
Age, mean (SD)	54.7 (10.4)	54.6 (10.3)
Male, n (%)	120 (88.9)	107 (81.1)
District in Karachi, n (%)		
Central	26 (19.3)	29 (22.0)
East	33 (12.4)	26 (19.7)
Korangi	18 (6.7)	8 (6.1)
Malir	9 (33.7)	19 (14.4)
South	28 (20.7)	21 (15.9)
West	21 (15.6)	29 (22.0)
Additional professional qualification,* n (%)	27 (20.0)	25 (18.9)
Years of experience, mean (SD)	30.1 (10.1)	30.1 (9.9)
Self-reported number of patients seen weekly, n (%)		
<25	40 (29.6)	38 (28.8)
26–50	65 (48.1)	60 (45.5)
51–75	17 (12.6)	15 (11.4)
>76	13 (9.6)	19 (14.4)
Self-reported number of meetings with pharmaceutical sales representatives weekly, n (%)		
0	15 (11.1)	13 (9.8)
<25	104 (77.0)	96 (72.7)
26–50	11 (8.1)	17 (12.9)
>51	5 (3.7)	6 (4.5)
Works at two or more practices, n (%)	21 (15.6)	24 (18.2)

* Membership of College of Physicians and Surgeons Pakistan (MCPS); Fellow of College of Physicians and Surgeons Pakistan (FCPS); Diplomate of American Board; Diploma; Membership of the Royal Colleges of Physicians UK (MRCP); Membership of Royal College of General Practice UK (MRC-GP).

In the control group, 52 private doctors (41.9%) agreed to accept incentives in exchange for prescribing promoted medicines, as compared with 42 (32.3%) in the intervention group (table 2). There was no evidence of an adjusted effect of the intervention on the primary outcome (OR 0.70 (95% CI 0.40 to 1.20), p=0.192). The majority of private doctors who agreed to accept incentives for prescribing promoted medicines opted for a financial incentive, such as cash or cheque payment, followed by clinical equipment or renovation (table 3). Of those participants who refused to accept incentives, the most common reasons were reluctance to work with franchise companies or already being fully committed to incentive agreements with other pharmaceutical companies (table 4).

**Table 2:** Effect of intervention on primary and secondary outcomes

Outcome	Intervention (n=135)	Control (n=132)	Unadjusted effect (95% CI)	P value	Adjusted* effect (95% CI)	P value
Primary†	n (%)	n (%)	OR		OR	
Acceptance of incentives (%)	42 (32.3)	52 (41.9)	0.66 (0.40 to 1.10)	0.113	0.70 (0.40 to 1.20)	0.192
Secondary†	Mean (SE)	Mean (SE)	Difference		Difference	
Knowledge of conflict of interest (score out of 3)	1.47 (0.09)	1.23 (0.09)	0.24 (0.003 to 0.48)	0.047	0.26 (0.02 to 0.50)	0.031
Knowledge of policy (score out of 4)	3.01 (0.07)	2.87 (0.08)	0.15 (−0.05 to 0.35)	0.148	0.13 (−0.08 to 0.33)	0.219
Attitude (score out of 7)	5.20 (0.09)	4.94 (0.12)	0.26 (−0.03 to 0.56)	0.078	0.30 (−0.01 to 0.60)	0.055

* Adjusting for physician’s age and sex, and district of practice.

† Eight doctors in the control arm and five doctors in the intervention arm were not accessible for the primary outcome assessment.

N, number of observations; n, number of events.;

**Table 3:** Types of incentives accepted or requested

Type of incentive	Intervention (n=135) (%)	Control (n=132) (%)
Payment in return for prescribing (eg, commission, cash or cheque)	29 (21.5)	34 (25.8)
Clinic equipment or renovation	6 (4.4)	11 (8.3)
Leisure trip or meal out with their family	3 (2.2)	5 (3.8)
No specific incentive agreed	1 (0.7)	2 (1.5)
Monthly lease payment for personal car	1 (0.7)	0 (0)
Two incentive types	2 (1.5)	0 (0)

**Table 4:** Reasons participants gave for declining to engage in incentive-linked prescribing deals

Reason	Intervention (n=83) (%)	Control (n=69) (%)
Does not make deals with any pharmaceutical companies	27 (32.6)	30 (43.5)
Not interested in engaging with franchise companies and/or already fully engaged with other companies	52 (62.6)	38 (55.0)
The clinic receptionist advised the standardised sales representative to leave	3 (3.6)	1 (1.4)
No reason provided	1 (1.2)	0 (0)

For the secondary outcomes, our intervention increased knowledge of conflict of interest arising from pharmaceutical incentivisation (intervention group mean score 1.47 vs control group 1.23); the adjusted mean difference between groups was 0.26 (95% CI 0.02 to 0.50, p<0.031) (table 2). There was no evidence of a difference in knowledge of policies related to ethical prescribing between groups (3.01 vs 2.87; adjusted mean difference 0.13 (95% CI −0.08 to 0.33, p<0.219). There was weak evidence of an impact of the intervention on attitude towards accepting incentives scores (5.20 vs 4.94; adjusted mean difference 0.30 [95% CI −0.01 to 0.60], p<0.055) (table 2).

Finally, our interviews indicated that although the intervention seminar content was remembered by participants, (perceived) pressure to generate sufficient income worked against ideas about doctors’ moral responsibility to avoid incentivisation by pharmaceutical companies. The interview results are elaborated on in a separate study.^31^ A sustained impact of the intervention also appeared to be limited by the ease with which doctors are able to engage in incentive-linked prescribing deals, owing to constant offers by pharmaceutical company representatives and no risk of any penalties from engaging in incentive-linked prescribing.

## Discussion

This study using a novel covert method to assess prescribing deals between doctors and standardised pharmaceutical sales representatives revealed how common such deals are: over 40% of doctors who were not exposed to our intervention agreed to incentives in exchange for prescribing medicines from a fictitious pharmaceutical company. Further, we found that our carefully designed emotive-educational intervention did not significantly reduce the proportion of private doctors agreeing to accept incentives in exchange for prescribing specified medicines, despite resulting in increased knowledge about ethical prescribing. Doctors who agreed to incentive-linked prescribing deals did so without any evidence of the quality of medicines being promoted within two interactions with our standardised sales representatives. Of those doctors who refused to accept incentives, the majority indicated interest in discussing incentive-linked prescribing deals with other pharmaceutical companies known to them, suggesting that the refusal was not related to a stance against incentivisation.

The evidence we have generated is consistent with other studies indicating that incentive-linked prescribing is widespread, that doctors play an active role in making deals with pharmaceutical sales representatives and that their practices are difficult to change.^8 32^ Our findings highlight an important barrier to evidence-based use of medical products and have implications for antibiotic stewardship since antibiotics were among the medicines doctors agreed to prescribe in exchange for incentives.

Our intervention likely had a limited impact because of the numerous systemic drivers of incentive-linked prescribing, including the profit generating motivations of private doctors; inappropriate relationships with the pharmaceutical industry being observed during clinical training; and pushing of attractive incentives by pharmaceutical companies.^19^ Taken together, available evidence indicates that clear policies for doctors and the pharmaceutical industry, with enforced penalties for non-compliance, alongside education and peer review, might be needed to change doctors’ practices.^33 34^ However, the impact of regulations that prohibit doctors from receiving gifts or sponsorship and that restrict interactions between pharmaceutical company representatives and doctors remains unclear.^3335^,^37^ Similarly, policies mandating disclosures about financial ties between health professionals and industry have gained traction in some countries, but without clear evidence of resultant changes in prescribing practice.^38 39^ Ultimately, if there is widespread support for incentive-linked prescribing deals from pharmaceutical companies and doctors themselves, substantial changes in practice are challenging to achieve.

### Limitations

Our study findings have important implications for clinicians, policy-makers and wider society, and we outline some methodological limitations here. As is expected of randomised trials, our geographical scope had to be confined, although to one of the most populous cities in the world. Reflecting the practising health workforce in Pakistan, our study population contained a small number of females; this prevented us from being able to explore gender differences in acceptance of incentives. Furthermore, because our formative research indicated that private doctors would be unlikely to leave their practices for long periods to avoid a loss of income, we had a short one-off in person seminar and designed reinforcement messages that could be delivered via letters and WhatsApp.

While our novel method was effective in assessing the behaviours of private doctors and may be applied to other contexts with insufficiently regulated pharmaceutical and private healthcare sectors, the standardised sales representatives operated under several constraints: they represented fictional pharmaceutical franchises and did not have free samples to offer; they lacked pre-established relationships with doctors; and they were limited to a maximum of two interactions with doctors for them to agree to or reject incentives. The proportion of doctors agreeing to an incentive-linked prescribing deal without these constraints could therefore be expected to be higher.

Finally, we acknowledge the potential for harm from results of this trial. Highlighting the prevalence of doctors making deals for prescribing with pharmaceutical industry representatives could unduly lower patients’ trust in doctors, possibly leading to the use other healthcare providers that are less qualified. Covert evaluations of doctors may influence their trust of researchers or regulators monitoring them. The findings of this study must therefore be shared sensitively and responsibly.

## Conclusion

To conclude, adding to the limited empirical evidence on deals between doctors and pharmaceutical companies, our findings call into question the impact of educational and emotional messages to dissuade doctors from engaging in incentive-linked prescribing deals with the pharmaceutical industry. Interventions targeting medical students or doctors who are in training (before incentivisation practices are established), interventions involving mandatory professional ethics training introduced by a regulator, and approaches to improve adherence to ethical marketing practices by pharmaceutical companies should be investigated. Despite complexities in measuring and addressing incentive-linked prescribing, it is nonetheless important to focus on this issue considering the conflicts of interest that characterise for-profit healthcare provision, which is expanding around the world.

## Supplementary material

10.1136/bmjgh-2024-016055 online supplemental file 1

10.1136/bmjgh-2024-016055 online supplemental video 1

## Data Availability

Data are available upon reasonable request.
