# Supplementary material for: Doctors taking bribes from pharmaceutical companies is common and not substantially reduced by an educational intervention: a pragmatic randomised controlled trial in Pakistan
Source: BMJ Glob Health. 2025 Jan 15;9(12):e016055. doi: 10.1136/bmjgh-2024-016055 (PMC11789533; doi:10.1136/bmjgh-2024-016055)
Supplement: online supplemental file 1 [file bmjgh-9-12-s001.pdf]

Supplementary materials

Questions on knowledge and attitudes (secondary outcomes)

**Knowledge of conflict of interest**<br>[Correct response in brackets]
A conflict of interest in medical practice is always associated with harm to the patient [False]
Doctors experience a conflict of interest only if they accept a financial incentive [False]
Doctors can experience a conflict between their personal interests and their patient's interests without taking any actions [True]
**Knowledge of national policies on physician engagement with pharmaceutical industry**<br>[Correct response show in brackets]
Pharmaceutical companies can directly pay for or reimburse the expenses of an individual doctor to attend a conference [False]
Interactions between pharmaceutical companies and doctors should not be dependent upon sales transactions or use or recommendation of the company's products [True]
As long as there are some educational activities at a scientific conference, it is fine for there to also be recreational activities, such as city tours and lavish meals [False]
Doctors may accept educational items, such as a medical textbook, from a pharmaceutical company if it serves a genuine educational function and is low cost [True]
**Attitude towards accepting different incentive types in return for prescribing targets**<br>[Correct response is 'Unacceptable' for all]
Sponsorship to attend a seminar internationally or nationally
A new air-conditioning unit or refrigerator for my clinic
Money, either cash or cheque
A nice meal out with my family or other doctors

A few small branded items, such as a pen, notepad, or calendar
A new car
Medical equipment that will benefit the doctors' patients, such as an ECG machine or stethoscope

*Adjusted for age and years of experience
